# “To Do or Not to Do, That Is the Question”, Surgery and Pregnancy

**DOI:** 10.3390/jcm11175095

**Published:** 2022-08-30

**Authors:** Salvatore Andrea Mastrolia

**Affiliations:** Department of Obstetrics and Gynecology, Ospedale Civile Umberto I, Via Ruvo, 108, 70033 Corato, Italy; mastroliasa@gmail.com

Whenever we associate the terms “pregnancy” and “surgery”, we tend to first think to cesarean sections.

However, there are many issues related to surgery and pregnancy, the first one being whether we have to perform non-obstetric surgery during pregnancy and what effect this can have on obstetric outcomes.

Non-obstetric surgical procedures during pregnancy represent a prominent fear of all surgeons. These are not common procedures due to (a) the intrinsic low incidence of the conditions requiring not deferrable surgery; and (b) the fact that during pregnancy, all attempts to avoid surgery using medical treatments are tried [1]; however, surgery is sometimes necessary, and an incidence of 0.75% has been reported [2]. Different types of surgeries have been performed during gestation. Of interest, the majority of the data come from appendectomies, which are considered to be safe procedures in all trimesters [3,4,5]. According to different studies, appendectomy rates are reported to range between 0.04 and 0.2% [6].

A multidisciplinary team including Obstetricians, Neonatologists, Anesthesiologists, and Surgeons should be involged to manage women requiring non-obstetrical surgical procedures during pregnancy [7]. Adapting anesthetic procedures and surgical techniques may be needed due to the physiological and anatomical changes related to pregnancy, as well as fetal concerns [8,9]. There is no evidence that in utero human exposure to sedative or anesthetic drugs might have an effect on the developing fetal brain, and there are no animal data showing an effect with exposure durations limited to less than three hours [7].

The choice of the surgical technique depends on the patient’s medical condition, gestational age, surgeon’s skills, and surgical goals and needs. Laparoscopy is considered, for the non-pregnant population, a standard of care in many cases if performed by a skilled surgeon. Of interest, laparoscopy offers the same advantages to the pregnant as to the non-pregnant woman (decreased analgesic use, a shorter hospitalization time, and faster recovery) [10] and can be performed safely during pregnancy [11]. The American College of Obstetricians and Gynecologists (ACOG) recommends that a pregnant woman should never be denied a necessary surgery or have that surgery delayed regardless of the trimester of pregnancy because this can adversely affect the pregnant woman and her fetus [7]. Moreover, due to the risk of preterm delivery when performing non-obstetric surgeries during pregnancy, corticosteroid administration for fetal lung maturity should be considered for women carrying preterm viable fetuses, and patients should be monitored in the perioperative period for symptoms or signs of preterm labor [7].

The second serious issue relates to how to avoid surgeries that should not be performed, such as surgical procedures involving the uterus reducing the potential negative effect of uterine surgery on subsequent pregnancies. In this second group of conditions, the efforts to reduce the incidence of a first cesarean section as well to increase the rate of trial of labor after cesarean (TOLAC) should be included. Cesarean delivery is the most common surgery worldwide, and in the United States, nearly one-third of pregnant women deliver by cesarean section [12]. Of the nearly 3.5 million women who deliver in the United States every year, 520,000 discuss whether to attempt a TOLAC or deliver by an elective cesarean. Repeated cesareans are responsible for a significant part of the cesarean delivery rate [13]. Over the last 20 years, we observed a reduction of women choosing to undergo a TOLAC, with the rate having declined from 28% in 1996 to 12% in 2015 [14].

A population-based study found that the risk of severe maternal morbidities associated with cesarean delivery (hemorrhage requiring hysterectomy or blood transfusion, uterine rupture, acute renal failure, shock, cardiac arrest, venous thromboembolism, sepsis, in-hospital wound disruption or hematoma, anesthetic complications, and assisted ventilation) compared to vaginal delivery was threefold higher in women undergoing a cesarean (2.7% versus 0.9%, respectively) [15]. In addition, there are concerns regarding the long-term risks associated with cesarean sections, with particular attention given to those associated with future pregnancies. Placental abnormalities, such as placenta previa and/or accreta spectrum in subsequent pregnancies show an increased incidence with each cesarean delivery. This incidence raises from 1% with one prior cesarean section to around 3% in women undergoing their third or more surgeries. Of great interest, after three cesarean deliveries, the risk that a placenta previa will be complicated by accreta spectrum conditions is nearly 40% [16].

In an effort to reduce the rate of primary cesarean deliveries, there is also an instrument represented by external cephalic version, to be performed in those women whose fetuses are in non-cephalic presentation. A breech presentation occurs in approximately 3–4% of term pregnancies [17], and there is a high cesarean delivery rate for breech presentations. An external cephalic version provides an option of reducing cesarean delivery rate, although the implementation of the maneuver varies, with an estimated 20–30% of eligible women not being offered the maneuver due to the absence of personnel with expertise [18].

In conclusion, surgery can be performed during pregnancy for non-obstetric reasons, while unnecessary obstetric surgeries that create a risk for subsequent pregnancies need to be avoided, putting all efforts into implementing an adequate counseling process for pregnant women and increasing the operators’ expertise in order to improve future outcomes.
